# Metagenomic approach reveals microbial diversity and predictive microbial metabolic pathways in Yucha, a traditional Li fermented food

**DOI:** 10.1038/srep32524

**Published:** 2016-08-31

**Authors:** Jiachao Zhang, Xiaoru Wang, Dongxue Huo, Wu Li, Qisong Hu, Chuanbiao Xu, Sixin Liu, Congfa Li

**Affiliations:** 1 College of Food Science and Technology, Hainan University, Haikou 570228, P. R. China

## Abstract

Yucha is a typical traditional fermented food of the Li population in the Hainan province of China, and it is made up of cooked rice and fresh fish. In the present study, metagenomic approach and culture-dependent technology were applied to describe the diversity of microbiota and identify beneficial microbes in the Yucha. At the genus level, *Lactobacillus* was the most abundant genus (43.82% of the total reads), followed by *Lactococcus, Enterococcus, Vibrio, Weissella, Pediococcus, Enterobacter, Salinivibrio, Acinetobacter, Macrococcus, Kluyvera* and *Clostridium*; this result was confirmed by q-PCR. PCoA based on Weighted UniFrac distances showed an apparent clustering pattern for Yucha samples from different locations, and *Lactobacillus sakei, Lactobacillus saniviri* and *Staphylococcus sciuri* represented OTUs according to the major identified markers. At the microbial functional level, it was observed that there was an enrichment of metabolic functional features, including amino acid and carbohydrate metabolism, which implied that the microbial metabolism in the Yucha samples tended to be vigorous. Accordingly, we further investigated the correlation between the predominant microbes and metabolic functional features. Thirteen species of *Lactobacillus* (147 strains) were isolated, and *Lactobacillus plantarum* (60 isolates) and *Lactobacillus pentosus* (34 isolates) were isolated from every sample.

The Li population is an indigenous settlement of the Hainan province, which is located in the southernmost region of China. Fermented foods have played an important role in the Li daily diet since ancient times, which has been attributed to their rich taste and nutrition^1^. Yucha is a typical traditional fermented food of the Li population, and it is made up of cooked rice and fresh fish. By adding some salt and hot pepper, the cooked rice and fresh fish are put into a porcelain jar, where they are pickled and ferment for at least one month in an anaerobic environment. Currently, Yucha is still the most popular supplementary food of the Li settlements, and nearly every Li family has the habit of Yucha production.

Traditional fermented foods such as Yucha in the Hainan province of China are common around the world, and most studies have focused on the diversity of microbiota and the isolation of beneficial microbes in them^2^. Drinks such as Airag and Tarag, which are mildly alcoholic, sour-tasting fermented drinks that are usually made from the raw milk of mares or camels, are very popular beverages that are traditionally produced in Mongolia, Kazakhstan, Kyrgyzstan, and some Central Asian regions of Russia^3^. By applying culture-dependent and culture-independent technology, Koichi identified 367 *Lactobacillus* and 152 yeast strains in Airag and Tarag, and the *Lactobacillus helveticus* and *Lactobacillus kefiranofaciens* were the predominant bacterial species^4^. A similar study was carried out to characterize Munkoyo, Chibwantu and Mabisi, which are typical non-alcoholic fermented beverages in Zambia^5^. By performing amplified ribosomal DNA restriction analysis (ARDRA), Sijmen described the microbial community structure and identified *Lactobacillus, Leuconostoc, Lysinibacillus* and *Bacillus* as the predominant genera in these Zambian fermented samples^5^. These studies showed that the microbial resources in traditional fermented foods all around the world were diverse and abundant and that species such as *Lactobacillus delbrueckii* subspecies *bulgaricus* and *Lactobacillus plantarum* exhibited extreme polymorphisms associated with geographical distribution^6^,^7^.

The Hainan province is located in tropical southern China, and it is separated from mainland China by the sea. The microbial resources are abundant and diverse in Hainan province because of its typical island-associated geographical features^8^. The Li population has inhabited the Hainan province for generations, and they have ample experience with the production of various fermented foods through the use of environmental microbes. Yucha represents the most popular traditional fermented food in the Li population, and it is thus the best model for investigating tropical microbial diversity and the source of beneficial microbes. To describe the microbial diversity in traditional fermented food for further selection and application of beneficial microbes, we investigated the Yucha samples collected from different Li settlements in the Hainan province by high-throughput sequencing combined with q-PCR and culture-dependent technology. The present study brings new discoveries for the field of microbial resources and creates new opportunities for humans to use these important and beneficial microbial resources.

## Results

### The determination of acidity and alpha diversity in Yucha samples

The results of the microbial alpha diversity and acidity measurements are shown in Table 1. The pH values were between 3.42 and 3.96 for most samples, and the TTA values were between 11.7 and 13.6 mL. We generated a dataset consisting of 602,379 filtered high-quality and classifiable 16S rRNA gene sequences, and an average of 25,099 sequences was obtained for each individual sample (range: from 22,451 to 28,068, Table S1). All sequences were clustered with representative sequences, and a 97% sequence identity cut-off was used. The number of OTUs per sample ranged between 72 and 140 (Table 1). The microbial alpha diversity, including the Simpson index, Chao1 index, Shannon index and observed number of species, was estimated using the QIIME^9^ platform (Table 1). There was no significant difference in alpha diversity among the Yucha samples collected from different locations.

### The microbial composition and core microbiota in Yucha samples

**Figure 1:**
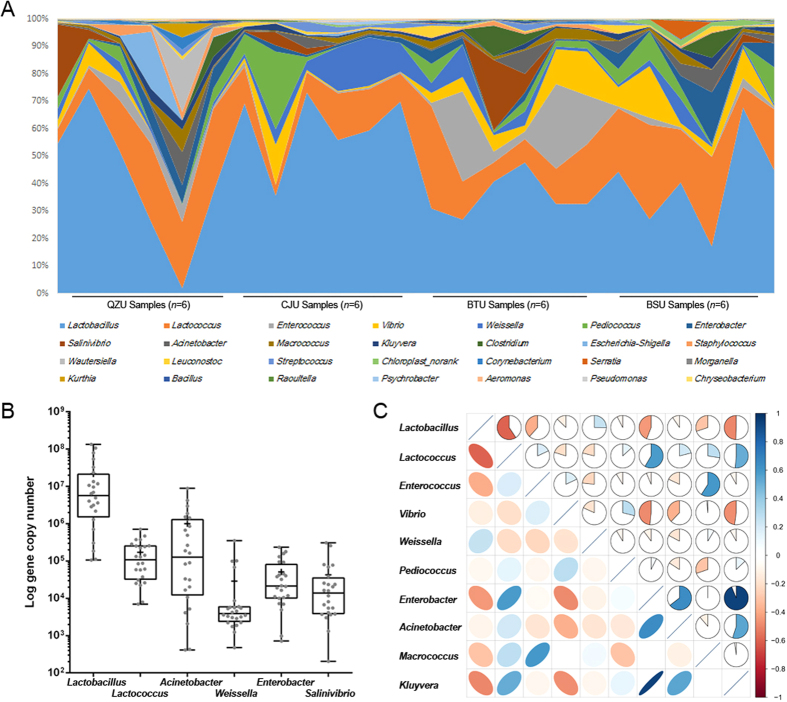
The composition of microbiota in Yucha samples. (**A**) Inter-individual variation in the proportion of major genus. (**B**) Box-plots showing the amounts of predominant bacteria as quantified using q-PCR. (**C**) Correlation matrix showing the Spearman’s rank correlation among the 10 core genus. The Spearman’s rank correlation coefficient ranges from 1.0 to −1.0, corresponding to a strongly positive to a strongly negative correlation.

At the genus level, *Lactobacillus* (Firmicutes phylum) was the most abundant genus (contributing to 43.82% of the total number of sequences), and the amounts of *Lactococcus, Enterococcus, Vibrio, Weissella, Pediococcus, Enterobacter, Salinivibrio, Acinetobacter, Macrococcus, Kluyvera* and *Clostridium* all exceeded 1% (Fig. 1A). Correlations among the above genera were determined based on Spearman’s rank correlation (Fig. 1C). A general negative correlation was found between *Lactobacillus* and other genera, and a general positive correlation was found between *Lactococcus* and other genera. Using genus-specific primers, we quantified the predominant microbiota in the Yucha samples (Fig. 1B). The amounts of *Lactobacillus*, *Lactococcus, Acinetobacter, Weissella, Enterococcus* and *Salinivibrio* genera were 7.33 ± 0.13, 5.23 ± 0.25, 5.99 ± 0.71, 4.46 ± 0.57, 4.71 ± 0.48 and 4.62 ± 0.57, respectively, in log-transformed 16S rDNA gene copy number per gram of sample.

### Differences in microbiota among Yucha samples from different locations

**Figure 2:**
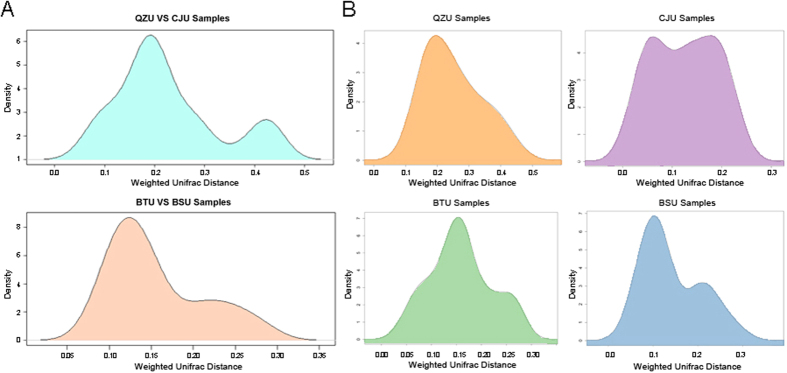
Differences in microbial composition among Yucha samples. (**A**) UniFrac distance distributions among samples in different locations. (**B**) UniFrac distances within each sampling group.

To explore the changes in the microbiota structure in the Yucha samples, we compared the Weighted UniFrac distances based on high-throughput sequencing data (OTU level) among Yucha samples collected from the Qiongzhong, Baisha, Baoting and Changjiang areas. As shown in the kernel density figure (Fig. 2A and Fig. S1), the UniFrac distance distributions of different groups were wide, which indicated a potential microbial structural difference among Yucha samples from different sampling locations. However, the UniFrac distances (OTU level) between samples within each group shown above were very small. Moreover, there was no significant clustering pattern for samples from the Qiongzhong area (Fig. 2B).

**Figure 3:**
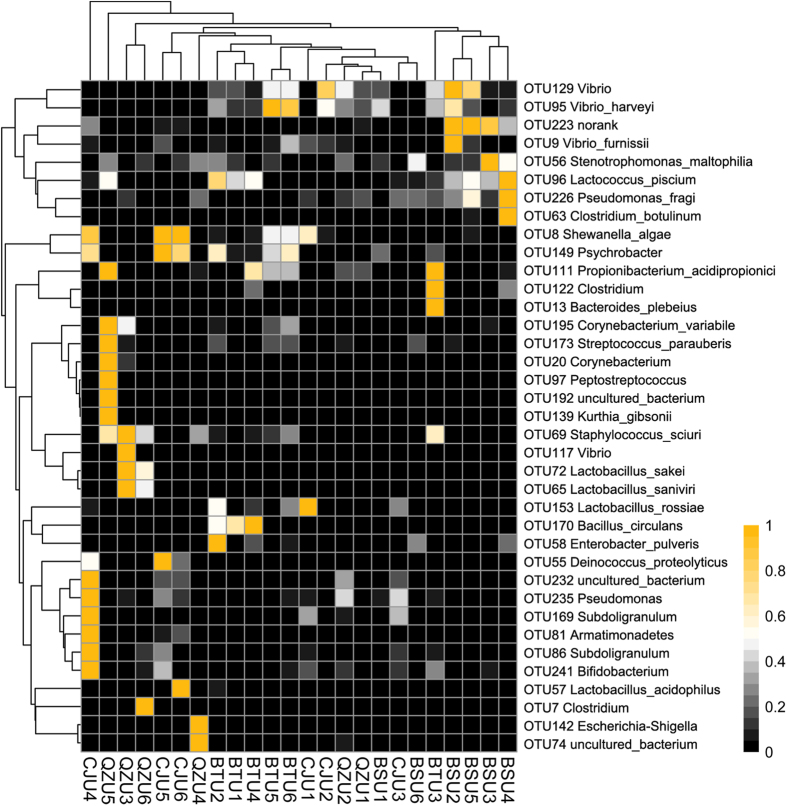
Heatmap constructed using the amount of significantly different OTUs among Yucha samples (OTUs normalized across samples) in different areas by Kruskal-Wallis test.

After confirming that there was a potential difference in the microbiota composition of the Yucha samples from different areas, we further identified differences in specific bacteria at the genus or species level. The OTU table was normalized according to the OTUs’ abundance across samples, and the significantly differences OTUs among samples in different sampling locations were clustered as the heatmap to give a better pattern across samples (Fig. 3). It can be observed that the species *Lactobacillus sakei*, *Lactobacillus saniviri*, *Corynebacterium variabile* and *Staphylococcus sciuri* represented OTUs (OTU 72, 65, 195 and 69) were enriched in samples from the Qingzhong area, though the amount of *Lactobacillus acidophilus* and *Lactobacillus rossiae* represented OTUs (OTU 57 and 153) was significantly lower than that found in samples from other areas. Meanwhile, the *Pseudomonas, Lactobacillus acidophilus, Bifidobacterium, Subdoligranulum* and *Lactobacillus rossiae* represented OTUs (OTU 235, 57, 241, 86 and 153) were enriched in samples from Changjiang area as well as the *Vibrio harveyi, Clostridium, Lactococcus piscium* and *Bacteroides plebeius* represented OTUs (OTU 95, 122, 96 and 13) were enriched in samples from Baoting and Baisha areas.

### Functional features of the microbiota isolated from Yucha samples

**Figure 4:**
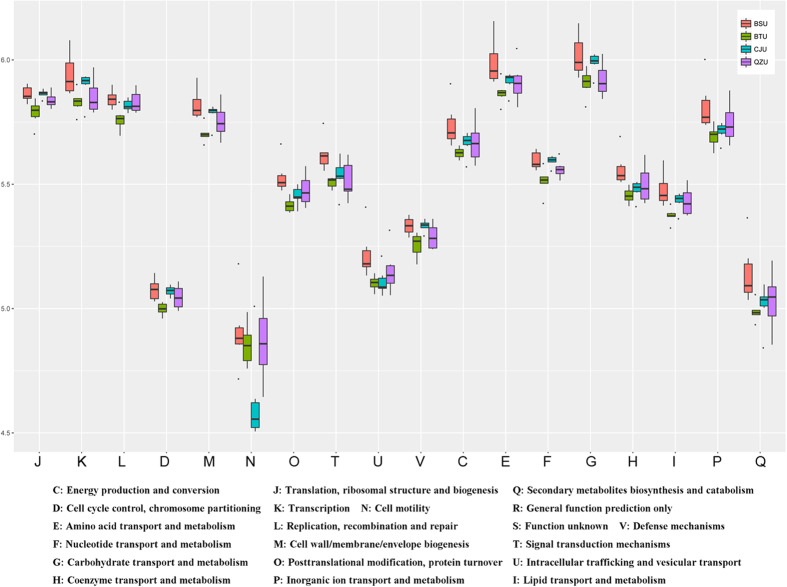
The microbial functional features in Yucha samples of different Li nationality settlements.

To better understand the important role of the microbiota present in Yucha samples, PICRUSt^10^ (phylogenetic investigation of communities by reconstruction of unobserved states) program was used to predict our 16S rRNA based high-throughput sequencing data and further analyze the data in the context of the Cluster of Orthologous Groups (COG) database. Using these methods, we obtained a microbial COG profile and correlated the microbial functional features with the important enzymes found in the Yucha samples (Fig. 4). It can be observed the metabolic functions were enriched in our samples, which implied that the microbial metabolism in the Yucha samples tended to be vigorous. And these functional features included: Energy production and conversion, Amino acid transport and metabolism; Nucleotide transport and metabolism; Carbohydrate transport and metabolism; Coenzyme transport and metabolism; Lipid transport and metabolism and Inorganic ion transport and metabolism.

### Concordance of core microbiota and metabolic functional features

**Figure 5:**
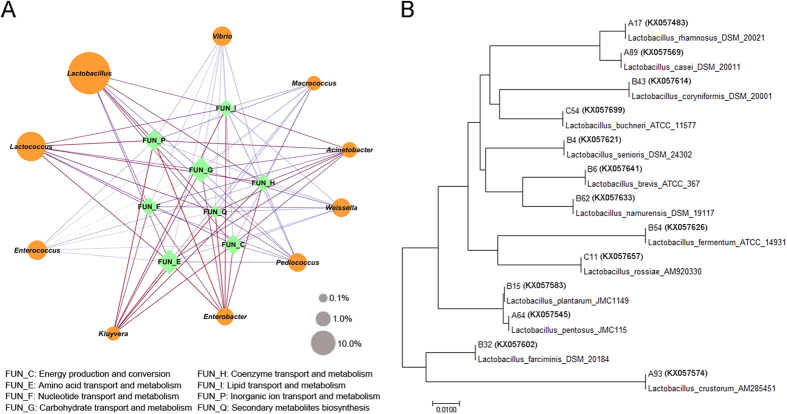
(**A**) Concordance of core microbiota and metabolic functional features. The edge width and color (red: positive and blue: negative) are proportional to the correlation strength. The node size is proportional to the mean abundance in the respective population. (**B**) Phylogenetic tree based on 16S rRNA gene sequences of *Lactobacillus* isolates.

Building on the core microbiota and the key functional features found in the Yucha samples, we further explored the correlation between the core microbiota and the metabolic functional features using the Spearman’s rank correlation coefficient. As shown in Fig. 5A, a general positive correlation can be found between *Lactococcus, Acinetobacter* and *Kluyvera* and all metabolic functional features. Meanwhile, *Vibrio* and *Weissella* were negative correlated with other functional features. Additionally, a significant positive correlation was found between *Lactobacillus*, which was the most abundant genus in the Yucha samples, and the metabolism of carbohydrates and nucleotides. Meanwhile, we calculated the contribution rate of the predominant genera to functional features related to microbial metabolism (Table 2). It was observed the genera of *Lactobacillus* and *Lactococcus* were the major functional contributors, which contributed 31.42% and 25.76% functional features related to the metabolism of amino acid and carbohydrate, respectively.

### Identification of *Lactobacillus* isolates

One hundred forty-seven *Lactobacillus* strains (Gram-positive and catalase negative) were isolated as pure cultures from the Yucha samples. Among these *Lactobacillus* isolates, eighty-nine strains were isolated from MRS agar, and fifty-eight strains were isolated from M17 agar. Molecular identification results and the phylogenetic tree based on 16S rRNA gene sequencing are shown in Table 3 and Fig. 5B. From the table, *Lactobacillus plantarum* (60 isolates) and *Lactobacillus pentosus* (34 isolates) isolated from every sample were the predominant *Lactobacillus* species found in Yucha samples. Moreover, *Lactobacillus farciminis* (16 isolates), *Lactobacillus brevi*s (10 isolates), *Lactobacillus rhamnosus* (6 isolates), *Lactobacillus casei* (6 isolates), *Lactobacillus coryniformis* (5 isolates), *Lactobacillus senioris* (2 isolates), *Lactobacillus namurensis* (2 isolates), *Lactobacillus fermentum* (2 isolates), *Lactobacillus buchneri* (2 isolates), *Lactobacillus rossiae* (1 isolate) and *Lactobacillus crustorum* (1 isolate) were isolated from Yucha samples collected from the settlements of the Li populations in the Hainan province of China. [Table t3]

## Discussion

In the present study, a combination of next-generation sequencing and culture-dependent technology was applied to investigate the microbial community of Yucha. It could be observed that the reads of *Lactobacillus* accounted for 43.82% of the total number of sequences; thus, this genus was the predominant one found in the Yucha samples; this result was also confirmed by q-PCR. Furthermore, thirteen species of *Lactobacillus* were isolated by a culture dependent method. Generally, lactic acid bacteria are a group of Gram-positive bacteria that are able to ferment glucose and lactose to lactic acid and are beneficial to human health^11^. *Lactobacillus* is a symbiotic bacterium found in fish intestinal microbiota, where it plays a key role in maintaining the balance in the gut micro-ecological environment^12^,^13^. As expected, considering the starting material (cooked rice and fish) and the processing of the Yucha samples, the abundant *Lactobacillus* found in our samples was likely rooted in the raw fish and the surrounding environment. In addition, the *Lactobacillus* proliferated and produced lactic acid, thereby creating an acid environment that inhibited the growth of other bacteria.

At the functional level, a general positive correlation between metabolic functional features (including energy production and conversion, amino acid transport and metabolism, nucleotide transport and metabolism and carbohydrate transport and metabolism) and core microbiota in the Yucha samples was highlighted. Fermented foods are a significant part of the daily diet of many people both in the Li populations and around the world. During the production of a fermented product, microorganisms transform raw material into a product with an increased value, generally by extending the shelf life of the raw materials and increasing the nutritional value of the product by improving the production of organoleptic attributes^14^,^15^. In the micro-ecological environment of Yucha, the cooked rice decomposition provided the carbon source for the growth of microbes^16^. Meanwhile, the fish decomposition generated amino acids that provided a nitrogen source^17^. Therefore, the microbes in the Yucha samples were vigorous.

Metagenomic approach has enabled exploration of microbial compositions in a range of traditional fermented foods while bypassing the need for cultivation, allowing the identification of a vast array of microorganisms never previously isolated in culture. By employing the pyrosequencing technology, Jung monitored changes in bacterial populations, metabolic potential, and overall genetic features of the microbial community during Kimchi fermentation^18^. It was observed the *Leuconostoc, Lactobacillus* and *Weissella* were the predominant genera in Kimchi, and a genetic profile characteristic of heterotrophic lactic acid fermentation of carbohydrates was revealed in the Kimchi microbiome. Compared with the microbial diversity between Kimchi and Yucha samples, the genera *Lactobacillus* and *Weissella* were predominant bacteria. However, the most abundant genus *Leuconostoc* in Kimchi was not detected in present research. The *Leuconostoc* was a common genus of lactic acid bacteria, and its abundant enzyme systems encoded by various genomic islands for fiber and polysaccharides fermentation were revealed by previous research^19^. Accordingly, the *Leuconostoc* was often isolated in vegetal fermented foods. The Yucha is made of cooked rice and fish (lack of vegetal material), so the micro-ecological environment was not suitable for the growth of *Leuconostoc.* In functional level, Jung focused on the microbial metabolic pathway of carbohydrate categories during Kimchi fermentation. And in Yucha samples, besides of the carbohydrate metabolism, the amino acid metabolism (Fig. 4) also kept in a high level because of the abundant fish materials added. Thus, not only region, but the raw materials used, may play a role in shaping the microbiome of the food items.

Additionally, the microbial beta diversity data (Fig. 3) imply that numerous different OTUs were represented in the Yucha samples collected from different regions, although *Lactobacillus* was the predominant genus in all samples. The most significant difference in microbial composition was found in the Yucha samples from the Qiongzhong and Changjiang areas, though the straight-line distance between the areas was not the longest among all areas. Geographically, the Qiongzhong area is located in the central region of the Hainan province, and it is surrounded by mountains, whereas the Changjiang area is located in the southwest of the Hainan province, near the sea. Hence, the geographical features between these areas are significantly different. In fact, the composition of microbes in fermented foods that originate from different geographical regions is influenced by many factors. The fermentation methods and the long history of sourdough production as well as the local environment play an important role in shaping the microbial species distribution^20^.

Hence, by combining new technologies like next generation sequencing and metagenomics, with conventional techniques like microbial culturing and q-PCR, we present an in depth profiling and characterization of microbiome of Yucha samples. The present study suggests Yucha to be a good source of beneficial microbes. Diverse microbial composition and high metabolic vigour (estimated using informatics approach) of the microbes present in Yucha may render them suitable for further exploration and appropriate applications by the scientific community.

## Materials and Methods

### Collection and chemical analysis of Yucha samples

In this study, 24 Yucha samples were aseptically collected from different Li settlement families (including the Qionghai, Changjiang, Baoting and Baisha areas) in the Hainan province of China. Samples were stored at 4 °C during the transfer to the laboratory and were analyzed within a few hours. The pH of the samples was measured, and the Total Titratable Acidity (TTA) values were determined according to previously described methods^21^.

### Sample processing and DNA extraction

Ten grams of Yucha sample was homogenized with 90 mL sterile NaCl solution (0.85%, w/v) to a homogenous suspension for 10 mins. DNA was extracted from the suspended material by using a QIAGEN DNA Mini-Kit (QIAGEN, Hilden, Germany) in combination with a bead-beating method^22^ according to the manufacturer’s instructions. The quality of the extracted DNA was assessed by 0.8% agarose gel electrophoresis, and the OD 260/280 was measured by spectrophotometry. All of the DNA samples were stored at −20 °C until further processing.

### PCR amplification, quantification, pooling and sequencing

The V3-V4 region of the 16S ribosomal RNA (rRNA) genes was amplified for each sample. A set of 6-nucleotide barcodes was added (Tab S1) to the universal forward primer 338F (5′-ACTCCTACGGGAGGCAGCA-3′) and the reverse primer 806R (5′-GGACTACHVGGGTWTCTAAT-3′)^22^,^23^. The PCR products were quantified with an Agilent DNA 1000 Kit using an Agilent 2100 Bioanalyser (Agilent Technologies, USA) according to the manufacturer’s instructions. The amplified products were pooled together in equimolar ratios, with a final concentration of 100 nmol/L each. These pools were sequenced by Illumina MiSeq using barcoded primers.

### Quantification of predominant genera in Yucha samples

The predominant bacteria of the Yucha samples were detected by quantitative PCR. Quantitative PCR was performed in an ABI Step-One detection system (Applied Biosystems). Based on the microbial abundance detected by the high-throughput sequencing, we chose the genera of *Lactobacillus, Lactococcus, Acinetobacter, Weissella, Enterococcus* and *Salinivibrio* as target microbes for quantification. The primers used for detecting the genera listed above and the reaction mixture (20 μl) were prepared as reported previously^24^.

### Isolation and identification of *Lactobacillus*

Ten grams of Yucha was homogenized with 90 mL sterile NaCl solution (0.85%, w/v) to a homogenous suspension, and a tenfold serial dilution was carried out. Then, 100 μl of each dilution was spread onto the surface of MRS (de Man, Rogosa and Sharp; Difco, Detroit, MI, USA) and M17 (Difco, Detroit, MI, USA) agar and incubated at 37 °C for two days to isolate *Lactobacillus*. Characteristic colonies were picked from each plate for morphology analysis. Isolates from the MRS and M17 agar were examined for a Gram reaction and catalase production. Cell morphology and motility were examined via the microscopic observation of cells grown in broth for 24 h (Olympus BX 40). DNA was extracted from *Lactobacillus* isolates according to Andrighetto *et al*.^25^.

The genomic DNA of each isolation was used as a template for PCR amplification, and the 16S rRNA gene was amplified by primers 27F (5′-AGAGTTTGATCCTGG CTCAG-3′) and 1492R (5′-GGTTACCTTGTTACGACTT-3′). The PCR procedure was performed as reported previously^26^. After the amplification, the PCR products (about 1,500 bp) were sequenced at the Shanghai Majorbio Corporation.

The sequences were visualized and manually aligned using the DNAMAN software (v5.0). Sequence homology was examined by comparing the obtained sequences with those in the NCBI database. The phylogenetic trees were constructed using the neighbour-joining distance method in the 6.0 version of the MEGA program. The confidence values of branches of the phylogenetic tree were determined using bootstrap analyses based on 500 random resamplings.

### Bioinformatic and Statistical analyses

The raw sequences were trimmed to remove the low-quality sequences. The sequences were filtered according to quality scores using the sliding window approach, in which sequences are trimmed when the average quality score over a 50-nt sliding window drops below 30. More than 85.3% sequence retained after filtering. Bioinformatic analyses were performed using QIIME (v1.6)^9^ on the extracted high-quality sequences. Briefly, the sequences were aligned using PyNAST^27^ and clustered under 100% sequence identity using UCLUST^28^ to obtain the unique V3-V4 sequence set. After representative sequences were selected, the unique sequence set was classified as an operational taxonomic unit (OTU) with a 97% threshold identity using UCLUST. The taxonomy of each OTU representative sequence was assigned using the Ribosomal Database Project (RDP)^29^ classifier with a minimum bootstrap threshold of 80%. A de novo taxonomic tree was constructed using a chimera-checked OTU representative set in FastTree^30^ for downstream analyses, including alpha and beta diversity calculations. To evaluate alpha diversity, the Shannon-Wiener and Simpson’s diversity indices and the Chao1 and rarefaction estimators were calculated. UniFrac^31^ metrics were calculated to evaluate beta diversity. Both weighted and unweighted calculations were performed prior to a principal coordinate analysis (PCoA). PICRUSt (Phylogenetic investigation of communities by reconstruction of unobserved states, v1.0) was used to predict the 16S rRNA based high-throughput sequencing data for functional features^10^.

All statistical analyses were conducted with the R programme (v3.3.0, https://www.r-project.org/). Based on the rarefied OTU subset, the relative abundances of taxa were compared by Kruskal-Wallis test. False discovery rate (FDR) values were estimated using the Benjamini-Yekutieli method to control for multiple testing^32^. PCoA analyses were performed in R using the ade4 package^33^. The correlation core OTUs were estimated by a Spearman rank correlation coefficient and visualized with a heatmap made in R using the “pheatmap” package.

## Additional Information

**Accession codes:** The sequence data reported in this paper have been deposited in the NCBI database (Metagenomic data: SRP072906; 16S rRNA: KX057487-KX057707).

**How to cite this article**: Zhang, J. *et al*. Metagenomic approach reveals microbial diversity and predictive microbial metabolic pathways in Yucha, a traditional Li fermented food. *Sci. Rep.* **6**, 32524; doi: 10.1038/srep32524 (2016).

## Supplementary Material

Supplementary Information

## Figures and Tables

**Table 1:** Sample information and microbial alpha diversity.

Yucha samples	Sampling location	pH value	TTA (mL)	Reads number
QZU (*n* = 6)	QiongZhong	3.96 ± 0.11	11.7 ± 0.3	25579 ± 1028
CJU (*n* = 6)	ChangJiang	3.74 ± 0.05	11.9 ± 0.5	24807 ± 2022
BTU (*n* = 6)	BaoTing	3.42 ± 0.47	13.6 ± 1.6	24282 ± 1487
BSU (*n* = 6)	BaiSha	3.51 ± 0.26	12.3 ± 0.4	25731 ± 3215
**Yucha samples**	**OTU number**	**Shannon index**	**Simpson index**	**Chao 1 index**
QZU (*n* = 6)	99 ± 15	2.34 ± 0.55	0.18 ± 0.02	137 ± 11
CJU (*n* = 6)	107 ± 33	2.15 ± 0.21	0.22 ± 0.05	130 ± 18
BTU (*n* = 6)	95 ± 18	2.39 ± 0.18	0.15 ± 0.02	119 ± 26
BSU (*n* = 6)	91 ± 21	2.27 ± 0.32	0.18 ± 0.04	116 ± 14

**Table 2:** The contribution rate (%) of the predominant genera to functional features related to microbial metabolism.

Feature	*Acinetobacter*	*Enterococcus*	*Lactococcus*	*Lactobacillus*	*Weissella*	Function	Function Description
C	5.3099	6.6048	8.7428	10.9115	0.0010	Metabolism	Energy production and conversion
E	4.5788	8.7794	13.2965	18.1203	0.0010	Metabolism	Amino acid transport and metabolism
F	2.3959	3.3928	5.4235	6.8999	0.0012	Metabolism	Nucleotide transport and metabolism
G	2.0890	6.1718	11.8069	13.9520	0.0013	Metabolism	Carbohydrate transport and metabolism
H	2.5603	2.6186	6.4481	8.6744	0.0007	Metabolism	Coenzyme transport and metabolism
I	3.1209	2.1415	4.7781	5.6961	0.0005	Metabolism	Lipid transport and metabolism
P	5.3020	7.1804	10.7633	12.4146	0.0008	Metabolism	Inorganic ion transport and metabolism
Q	2.2389	0.8279	5.5534	1.9064	0.0021	Metabolism	Secondary metabolites biosynthesis

**Table 3 t3:** Identification of *Lactobacillus* isolates based on 16S rDNA sequencing.

Identification	Samples			
	QZU	CJU	BTU	BSU
*Lactobacillus plantarum*	21/60	12/60	17/60	10/60
*Lactobacillus pentosus*	9/34	6/34	7/34	12/34
*Lactobacillus farciminis*	7/16	4/16	1/16	4/16
*Lactobacillus brevis*	3/10	—	5/10	2/10
*Lactobacillus rhamnosus*	2/6	4/6	—	—
*Lactobacillus casei*	3/6	1/6	—	2/6
*Lactobacillus coryniformis*	—	—	2/5	—
*Lactobacillus senioris*	1/2	—	1/2	—
*Lactobacillus namurensis*	—	1/2	—	1/2
*Lactobacillus fermentum*	1/2	1/2	—	—
*Lactobacillus buchneri*	1/2	—	1/2	—
*Lactobacillus rossiae*	1/1	—	—	—
*Lactobacillus crustorum*	1/1	—	—	—
