# Supplementary material for: Metagenomic approach reveals microbial diversity and predictive microbial metabolic pathways in Yucha, a traditional Li fermented food
Source: Sci Rep. 2016 Aug 31;6:32524. doi: 10.1038/srep32524 (PMC5006176; doi:10.1038/srep32524)
Supplement: Supplementary Information [file srep32524-s1.pdf]

Metagenomic approach reveals microbial diversity and predictive microbial metabolic pathways in Yucha, a traditional Li fermented food

Jiachao Zhang, Xiaoru Wang, Dongxue Huo, Wu Li, Qisong Hu, Chuanbiao Xu, Sixin Liu, Congfa Li*

The supplementary information including Table S1 and Figure S1

Table S1 The detailed of sequencing information in present research

Sample Name	Barcode	Sequences	Bases (bp)	Average Length (bp)
**QZU1**	ACGTCG	26636	11978131	449.7
**QZU2**	GCGTCT	23834	10721289	449.83
**QZU3**	ACGTGA	26690	12002644	449.71
**QZU4**	CCGTGC	27994	12591088	449.78
**QZU5**	GCGTGT	23676	10606551	447.99
**QZU6**	ACTACA	24646	11041888	448.02
**CJU1**	GCTACG	25334	11394688	449.78
**CJU2**	CCTACT	26319	11841518	449.92
**CJU3**	GCTAGA	22451	10097414	449.75
**CJU4**	ACTAGC	25000	11223094	448.92
**CJU5**	CCTAGT	24663	11088415	449.6
**CJU6**	GCTATC	25076	11279783	449.82
**BTU1**	CGAGCA	24982	11238509	449.86
**BTU2**	AGAGCG	23491	10566724	449.82
**BTU3**	GGAGCT	23060	10309237	447.06
**BTU4**	AGAGGA	23500	10552342	449.04
**BTU5**	GGAGGT	27116	12204493	450.08
**BTU6**	CGAGTC	23543	10591932	449.9
**BSU1**	GGAGTG	24550	11046171	449.95
**BSU2**	GGCTCG	25214	11333031	449.47
**BSU3**	AGCTCT	23987	10779060	449.37
**BSU4**	GGCTGA	28068	12557767	447.41
**BSU5**	AGCTGC	24955	11211338	449.26
**BSU6**	AGTCCA	27612	12424918	449.98

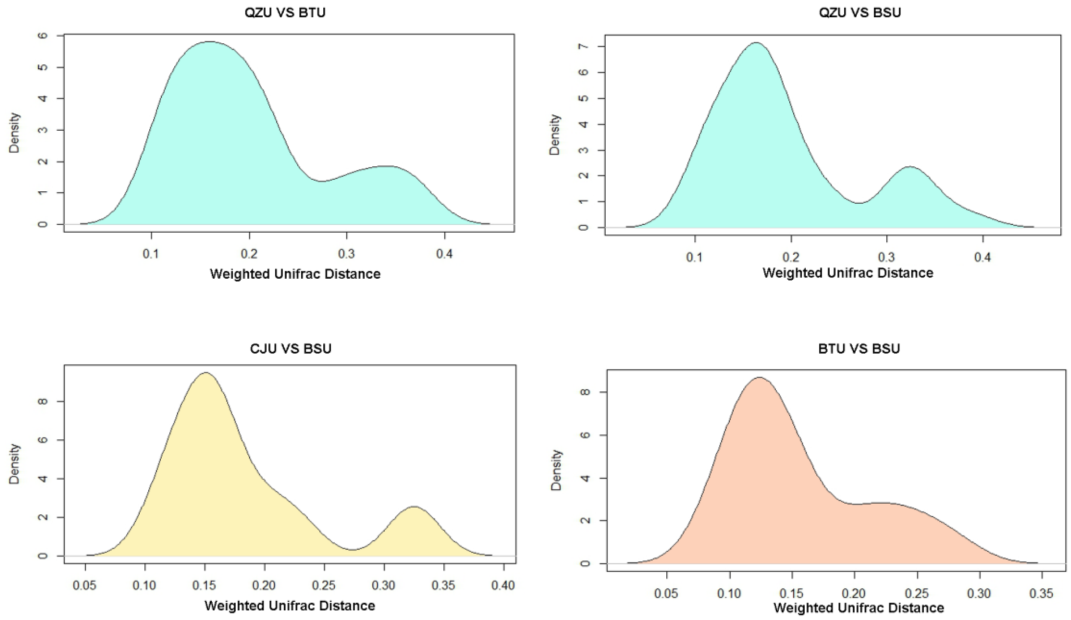

Figure S1 Weighted UniFrac distances between different sample groups.
